# A Neutrophil Proteomic Signature in Surgical Trauma Wounds

**DOI:** 10.3390/ijms19030761

**Published:** 2018-03-07

**Authors:** Sander Bekeschus, Jan-Wilm Lackmann, Denis Gümbel, Matthias Napp, Anke Schmidt, Kristian Wende

**Affiliations:** 1 Leibniz-Institute for Plasma Science and Technology (INP Greifswald), ZIK Plasmatis, Felix-Hausdorff-Str. 2, 17489 Greifswald, Germany; jan-wilm.lackmann@inp-greifswald.de (J.-W.L.); anke.schmidt@inp-greifswald.de (A.S.); kristian.wende@inp-greifswald.de (K.W.); 2 Department of Trauma, Reconstructive Surgery and Rehabilitation Medicine, Greifswald University, Medical Center Ferdinand-Sauerbruch-Str., 17475 Greifswald, Germany; denis.guembel@uni-greifswald.de (D.G.); nappm@uni-greifswald.de (M.N.)

**Keywords:** chaperones, damage-associated molecular patterns, heat-shock proteins, mass spectrometry, matrix metalloproteinase, peptidases, post-translational modifications, redox regulation

## Abstract

Non-healing wounds continue to be a clinical challenge for patients and medical staff. These wounds have a heterogeneous etiology, including diabetes and surgical trauma wounds. It is therefore important to decipher molecular signatures that reflect the macroscopic process of wound healing. To this end, we collected wound sponge dressings routinely used in vacuum assisted therapy after surgical trauma to generate wound-derived protein profiles via global mass spectrometry. We confidently identified 311 proteins in exudates. Among them were expected targets belonging to the immunoglobulin superfamily, complement, and skin-derived proteins, such as keratins. Next to several S100 proteins, chaperones, heat shock proteins, and immune modulators, the exudates presented a number of redox proteins as well as a discrete neutrophil proteomic signature, including for example cathepsin G, elastase, myeloperoxidase, CD66c, and lipocalin 2. We mapped over 200 post-translational modifications (PTMs; cysteine/methionine oxidation, tyrosine nitration, cysteine trioxidation) to the proteomic profile, for example, in peroxiredoxin 1. Investigating manually collected exudates, we confirmed presence of neutrophils and their products, such as microparticles and fragments containing myeloperoxidase and DNA. These data confirmed known and identified less known wound proteins and their PTMs, which may serve as resource for future studies on human wound healing.

## 1. Introduction

Wound healing is the essential process to protect the body from further environmental insults after trauma. It is segmented into discrete phases, namely hemostasis, inflammation, proliferation, and remodeling [1]. The inflammatory phase is central in steering healing into either a physiological or a pathological course. This phase is characterized by swift neutrophil influx, followed by later immigration of macrophage [2]. To oppose any invading pathogen expected during tissue injury, a pro-inflammatory milieu dominates that is controlled via chemokines and cytokines [3]. Once these pro-inflammatory setting is lacking sustained stimuli, e.g., due to clearance of bacteria, the mediator profile switches [4]. This causes macrophage re-programming towards an anti-inflammatory, pro-wound healing phenotype [5]. As consequence, keratinocyte and fibroblast influx is promoted, and the wound is sealed and remodeled [6].

Non-healing wounds and ulcers are a major burden for patients and western health care systems alike [7]. Molecularly, these wounds do not progress from the pro to the anti-inflammatory phase but stay in between them [8]. This is often coined “chronic” inflammation; i.e., an ongoing inflammatory phase that is attenuated but not resolved [9]. Chronic wounds are thought to be a consequences of either endogenous factors, such as impaired angiogenesis as seen in diabetic patients, or exogenous factors, such as excessive presence of pathogens [10]. In both cases, the wounds display distinct molecular signatures that are either a consequence or the driver of impaired healing [11,12,13]. To differentiate between features of pathological healing being hen or egg, studies on physiological healing greatly enhance the understanding of this highly complex, multi-facetted process [14]. Especially wound fluid has added to the understanding of healing in the past as it contains material from wound-resident cell such as neutrophils [15].

Neutrophils are the dominant cell type in inflammation as well as chronic wounds [16]. These cells are armed with an arsenal of molecules toxic to pathogens and host cells [17]. This includes the release of a number of enzymes with proteolytic and antimicrobial activity [18]. Neutrophils are also capable of release extracellular traps; antimicrobial DNA decorated with inflammatory enzymes [19]. Neutrophils have been observed to produce and release microparticles [20]; small double-membraned vesicles encapsulating signaling and enzymatic proteins, small molecules, and messenger RNA [21]. Importantly, the granulocytes are major producers of reactive oxygen and nitrogen species (ROS/RNS) [22]. Two major enzymes are superoxide-producing Flavin reductase (NADPH) oxidases and hypochlorous acid-generating myeloperoxidase [23]. Acute wound healing in rats has been observed to contain peak concentrations of 200 µM hydrogen peroxide [24]. This molecule is a major chemoattractant for neutrophils [25]. Consequently, it has long been proposed that wound healing is subject to redox control [26].

The aim of this study was to investigate wound exudates of acutely healing wounds on a cellular and protein level. Finding and confirming that large parts of cells in exudates are neutrophils as shown by surface marker profiles, we also identified neutrophil-derived small particles in our samples. Adding to and largely confirming a previous study [27], proteomic analysis of protein targets as well as their post-translational modifications (PTMs) revealed a in part unreported signature in the exudates of healing wounds.

## 2. Results

### 2.1. Proteomics of Wound Exudates

**Figure 1:**
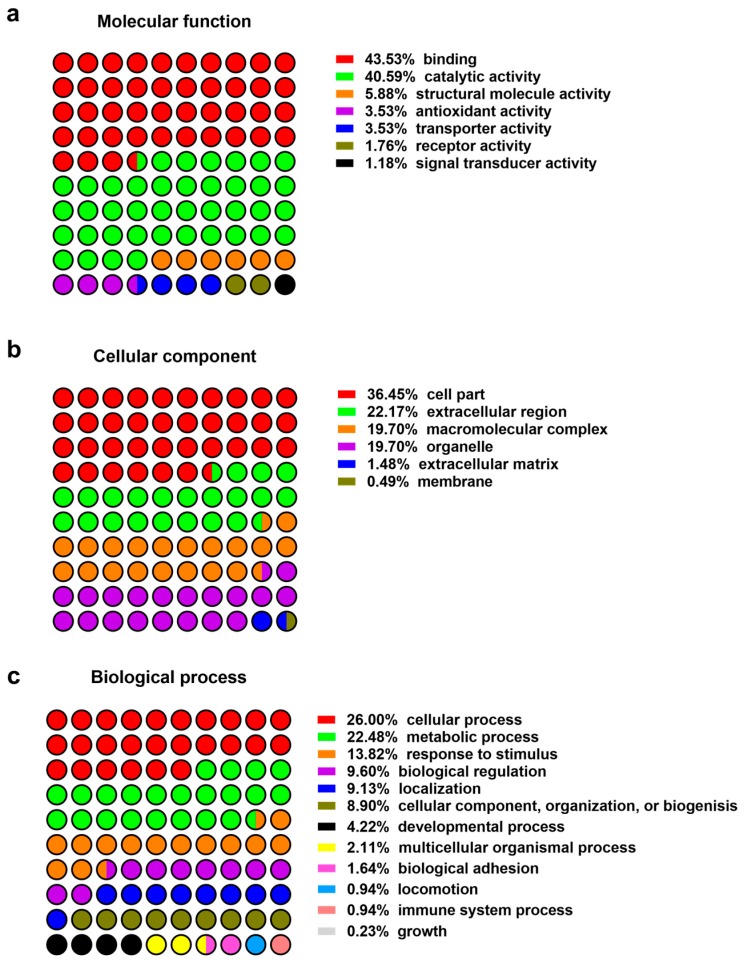
Protein classification: (**a**) Molecular function; (**b**) cellular compartment; (**c**) biological process.

**Figure 2:**
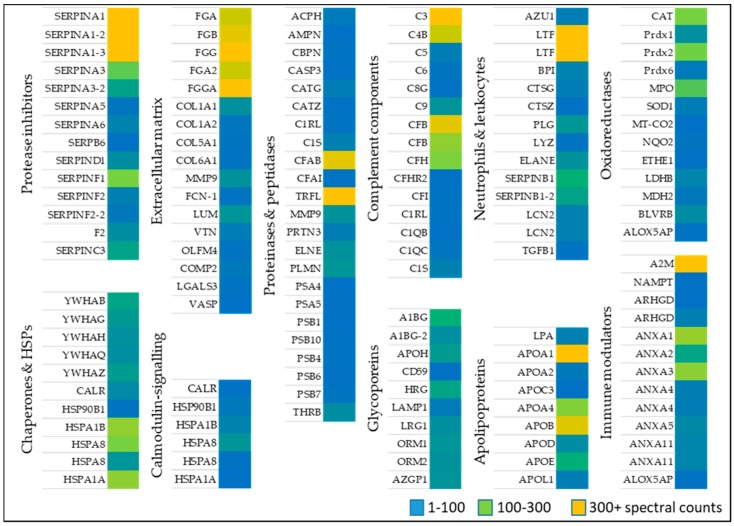
Relative abundance of high confidence proteins as determined in the pooled wound fluid samples by liquid chromatography/mass spectrometry spectral counts.

Identified proteins from wounds identified in patients (Table 1) were classified based on their function, compartment, and biological process (Figure 1). Alternatively, proteins were sorted based on their role in wound healing, e.g., activation of anti-oxidant factors such as oxidoreductases (Table 2), immune modulators, chaperone and heat shock proteins (Table 3), neutrophil and leukocytes associated factors (Table 4), extracellular matrix proteins (Table 5) as well as proteinases and peptidases (Table 6). An overview of the relative abundances of these proteins is given in Figure 2.

#### 2.1.1. Oxidoreductases

Anti-oxidative stress factors counteract oxidative stress present during wound healing. A large set of proteins belonging to anti-oxidant down-stream signaling response such as catalase (CAT) several peroxidases (PRDX1, 2, 6, MPO), and superoxide dismutase (SOD1) [28,29,30] was identified in wound fluids. Oxidoreductases such as cytochrome C oxidase (MT-COX2), the terminal enzyme in the mitochondrial respiratory chain, catalyze the reduction of oxygen for energy recovery [31] and its presence in wound fluids prevents an excessive inflammatory response [32]. l-lactate dehydrogenase (LDHB), a marker of tissue destructive microenvironment and hemolysis [33], was also identified. Dehydrogenases represents an additionally protein class of oxidoreductases, of which malate dehydrogenase (MDH2), and flavin reductase (NADPH; BLVRB) was detected (Table 2 and Figure 2). These data indicate the presence of enzymes of the anti-oxidative defense system in wound fluids obtained from the traumatic acute wounds.

#### 2.1.2. Immune Modulators, Chaperones, and Heat Shock Proteins

Several members of the complement factor family (Table A1) as well as numerous members of the S100A protein family (e.g., S100A4, 6, 8, 9, 11, S100P) were found in the exudates of traumatic wounds (Table 3 and Figure 2). Chaperone and heat shock proteins (HSP) are inducible stress proteins promoting wound closure by recruitment of dermal fibroblasts in late stages of wound repair [34]. We identified several HSP family members such as endoplasmin (HSP90B1), HSPA1A, HSPB, and HSP8. Moreover, calreticulin (CALR) and different 14-3-3 protein family members were detected in wound fluids (Table 3, middle part). The detection of α2-macroglobulin (A2M), which is mainly synthesized by macrophages and fibroblasts, suggest a functional inhibition of an enormous variety of proteinases. [35] Additionally, nicotinamide phosphoribosyltransferase (NAMPT) enables NAD^+^ biosynthesis, and functions as cytokine that promotes B cell maturation as well as inhibition of neutrophil apoptosis [36]. Two members of the Guanosine-5′-triphosphate (GDP)-dissociation inhibitors of Rho proteins (ARHGD1/2) were identified regulating GDP/Guanosine-5′-triphosphate (GTP) exchange and activating oxygen superoxide-generating NADPH oxidase of phagocytes [37]. Based on our proteomic approach, the abundance of several annexin family members (ANX1, 2, 3, 4, 5, 11) was further shown (Table 3, last part) confirming previous results provided by novel proteomic methodology [27].

#### 2.1.3. Neutrophil and Leukocytes Associated Factors

Numerous immune modulators such as lactotransferin (LTF), azurodicin (AZU1), bactericidal permeability-increasing protein (BPI) and lipocalin (LCN2) were identified (Table 4 and Figure 2), which are often associated with host defense against a broad range of microorganisms, immune response, anti-inflammatory activity and regulation of cellular growth as well as differentiation. A balance of the presence of neutrophil elastase (ELANE), inhibitors of leukocyte elastase (SERPINB1) and cathepsin G and Z (CTSG/Z) were found suggesting a strong regulation of a variety of proteolytic events important to tissue repair [27]. Further results also indicate an increased existence of protease inhibitors in the healing wound fluid (Table A2). Additionally, a proteolysis-activated plasminogen (PLG) and an antimicrobial enzyme lysozyme (LYZ) were detected in wound exudates indicating an activated stage of proteases in cellular processes such as wound healing and of innate immune system, respectively. Moreover, the anti-inflammatory transforming growth factor B1 (TGFβ1) activates macrophages and regulates a variety of cellular functions including cell proliferation, differentiation, and apoptosis, which are important during the different stages of wound healing.

#### 2.1.4. Extracellular Matrix Proteins

The extracellular matrix (ECM) is predominantly formed by collagen and is an essential component of the skin among fibroblasts, keratinocytes, endothelial cells, and immune cells [38]. Sixteen ECM molecules were obtained including several collagens (COL1A1, 2, 5, 6), fibrinogens (FGA, FGB, FGG) and oligomeric lectins consisting of both, collagen-like stretches and fibrinogen (e.g., FCN1) [39]. All proteins may function as hallmarks and biomarkers of newly generated granulation tissue and provisional wound matrix (Table 5 and Figure 2). Furthermore, lumican (LUM), vitronectin (VTN), olfactomedin-4 (OLFM4), and cartilage oligomeric matrix protein 2 (COMP2) were identified in wound fluids regulating tissue repair, collagen fibril organization and formation [40]. Galectin 3 (LGALS3) and the vasodilator-stimulated phosphoprotein (VASP), which were also found in wound fluids, are recruit to cell-cell junctions at the wound edge [41].

#### 2.1.5. Proteinases

Ubiquitous intracellular peptidases belonging to the proteasome (7 proteins: PSMAs/PSMBs; Table 6 and Figure 2) and caspase 3 (CASP3), indicate active inflammatory cellular processes [42] and show that cellular cytoplasmic fractions are present in the collected samples. The serine protease Cathepsin G (CATG) plays an integral part in immune response and inflammatory processes, being associated to NETs [18]. Aminopeptidase N (AMPN) is a peptidase with broad specificity, playing a role in MHCII presented antigen cleavage and in angiogenesis [43]. Apolipoprotein (APOA) inhibiting tissue-type plasminogen activator 1 and matrix metalloprotease 9 (MMP9) indicate a controlled ECM modulation and leukocyte migration [44]. Moreover, presence of prothrombin indicates the presence of blood in the wound bed [45].

#### 2.1.6. Post-Translational Modifications

Besides general annotation of proteins by mass spectrometry (MS), several chemical modifications were searched for in the generated data sets (Table A3). Numerous cysteine oxidations were observed: both cysteine sulfenic acid and, predominantly, cysteine sulfonic acid. Most single oxidations were found at methionines forming methionine sulfoxide, as cysteine sulfenic acid is both partially reduced by Dithiothreitol (DTT) and instable during ionization. In total, oxidative modifications were the most common observed changes compared to nitrosative modifications. Most nitrosative modifications were found to be tyrosine nitration as well as very low amounts of nitrosated cysteine. Additionally, carbamidomethylations were introduced during the reduction/alkylation work step during sample preparation.

### 2.2. Manually Obtained Wound Material

**Figure 3:**
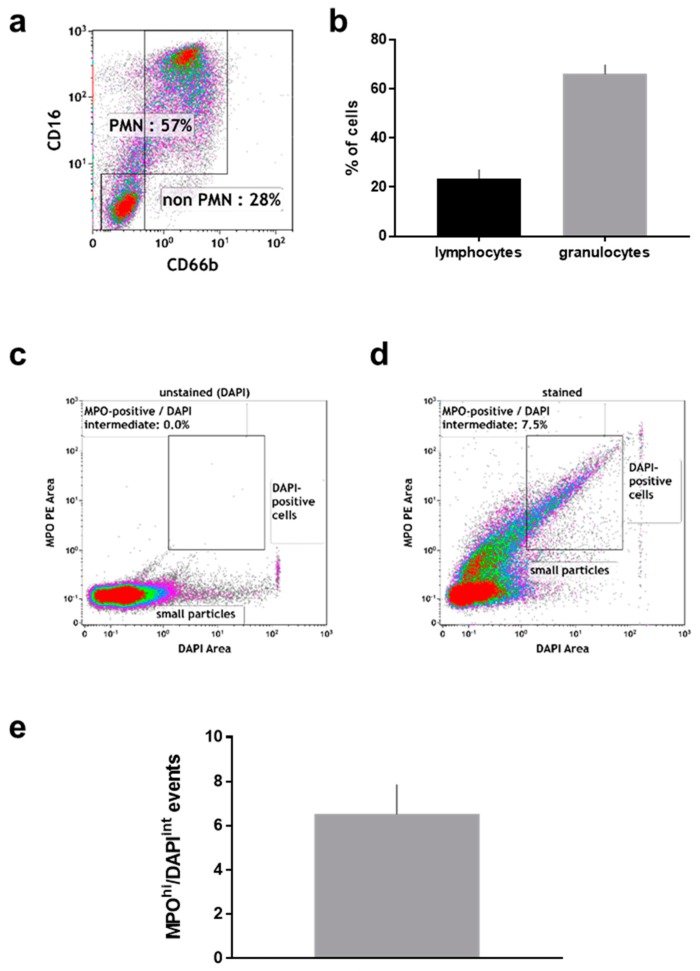
Flow cytometric analysis of cell pellets derived from manually removed wound material. (**a**) representative dot plot and (**b**) percent of CD16^+^/CD66b^+^ (neutrophils) and CD16^−^/CD66b^−^ cells among CD45^+^/DAPI^+^ cells; (**c**) pelleted material stained with DAPI only; (**d**) pelleted material stained against DAPI and MPO; (**e**) quantification of events staining positive for MPO and intermediate for DAPI. Data are from 6–8 patients.

**Figure 4:**
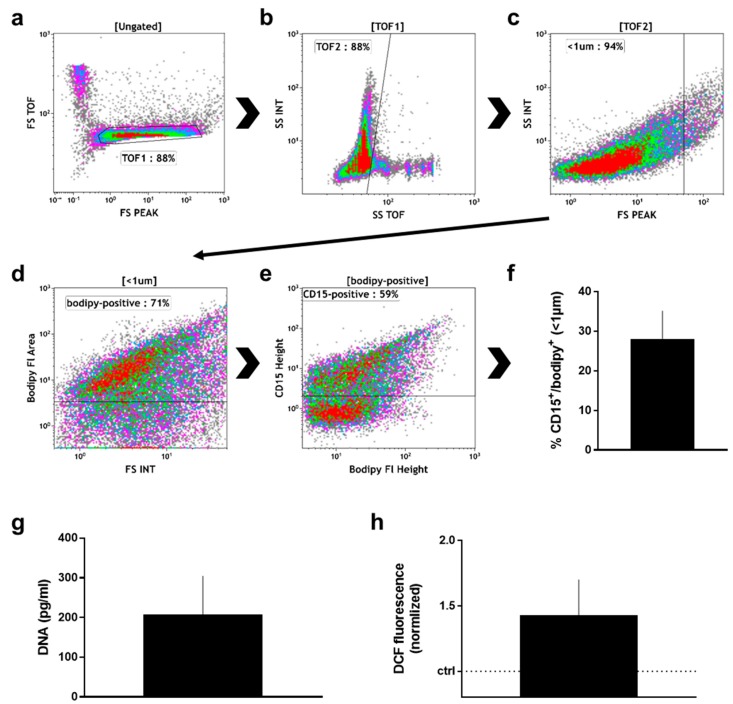
Supernatant analysis of manually removed wound material. (**a**) time of flight (TOF) gate in forward scatter (FS) height against FS TOF; (**b**) TOF gating analogous to (**a**); (**c**) size discrimination of small particles as predetermined with beads; (**d**) gate of membrane-positive particles as predetermined with unstained material; (**e**) discrimination between CD15^+^ and CD15^−^ microparticles as predetermined with unstained particles; (**f**) quantification CD15^+^ particles among all samples; (**g**) DNA content in supernatants as determined with sytox green against a known standard; (**h**) DCF fluorescence of wound material supernatant; data are one representative (**a**–**e**) or mean + S.E. of 8 (**f**), 3 (**g**), and 2 (**h**) samples.

After the wound sponge was removed, from which the material for mass spectrometry analysis was obtained, a swab was used to obtain additional wound material. From this, pelleted material as well as supernatant were analyzed. Within the pelleted material, the percentage (Figure 3a) of neutrophils (CD16^+^/CD66^+^) and non-neutrophils (CD16^−^/CD66^−^; mostly lymphocytes) among CD45^+^/DAPI^+^ cells was calculated (Figure 3b). There was about four times more neutrophils than non-neutrophils in the pelleted fraction. Neutrophils exposed to stimulating agents such as wound-resident bacteria are known to generate sticky DNA decorated with antimicrobial proteins such as myeloperoxidase (MPO), so-called neutrophil extracellular traps (NETs) [19]. To identify such structures, the pelleted material was stained for DNA (DAPI; Figure 3c), and MPO (Figure 3d). A substantial percentage of double-positive events among all acquired events was identified by flow cytometry in the pelleted fraction (Figure 3e). Another feature of eukaryotic cells in general and neutrophils in particular is the release of small vesicles carrying cargo protein and RNA for cell-cell communication and modulation of the tissue microenvironment, so-called microparticles [46]. To identify these small (<1 µm) membraned vesicles in the supernatant of wound material, we applied a stringent flow cytometry gating and staining strategy, tightly discriminating for doublets in the forward scatter (Figure 4a), side scatter (Figure 4b), and size exclusion (<1 µm; Figure 4c) using flow beads. Applying a membrane-stain, we were able to discriminate non-membraned events from microparticles (Figure 4d). Among microparticles, we tested for the percentage of neutrophil-derived CD15^+^ particles (Figure 4e) that were markedly present in the wound material supernatants (Figure 4f). As DNA^+^/MPO^+^ events were identified in the pelleted fraction (Figure 3d), we assayed for DNA (Figure 4g) and oxidants (Figure 4h) in the supernatant as well and found both to be increased.

## 3. Discussion

This study was conducted to better understand the proteomic signature paralleling physiological wound healing. While previous studies have identified numerous targets important in this process [47,48,49,50], we also identified so far less reported targets as potential biomarkers that contribute to cell growth, inhibit excessive inflammation, and support matrix as well as granulation tissue formation in normal skin repair. Our findings were complemented by data analysis of protein post-translational modifications and immune-focused analysis of wound exudates by flow cytometry.

Reactive oxygen species (ROS) and wound oxygenation are crucial processes in wound repair, and the oxygen-dependent redox-sensitive signaling represent an integral component of the healing cascade [51]. In wound healing, the initiating and activating of ROS-dependent signaling cascades that promote cellular responses are indispensable. We found indirect evidence of ROS in wound fluids and especially neutrophil-derived oxidants were reported to be crucial in the resolution of inflammation [52]. ROS mainly arise from metabolism via the mitochondrial electron transport chain [53] and a number of oxidases in phagocytes [54]. By proteomics, we identified neutrophil myeloperoxidase (MPO), a producer of potent hypochlorous acid [55]. By flow cytometry, we confirmed the presence of MPO not only in neutrophils from wounds but also on fragments that co-stained for DNA, suggesting the presence of neutrophil extracellular traps (NETs) in wound fluids. These sticky microbicidal DNA-extrusion are only poorly described in wound healing, despite the well-reported abundance of neutrophils in wounds [10]. NET formation is redox-regulated via MPO [56], and NETs form in response to microorganisms [57] and oxidants [58]. NETs were reported to be detrimental in diabetic foot healing [59], can be counteracted in wounds via DNAse treatment [60], and are potent ROS producers themselves [61,62,63]. ROS can be used as a signal for cell proliferation in which oxidoreductases are involved by regulating the redox state of proteins [64]. Among them, we found peroxiredoxins (PRDX1, 2, 6) in wound fluids suggesting a strong control of the wound healing-induced peroxide level [65]. In contrast to our proteomic analysis, PRDX2 was previously detected in exudate obtained from non-healing wounds only [27]. Nevertheless, due to the presence of further oxidoreductases (e.g., SOD1, MT-CO2), acute inflammation is accompanied by metabolic changes and a suppression of mitochondrial respiration. Thus, significant alterations in the anti-oxidant profile accompanied by the presence of an arachidonate 5-lipoxygenase-activating protein (ALOX5AP), a lipid mediator of inflammation [66], and numerous other apolipoproteins (Table A2) may be contributory to healing of traumatic wounds. It was shown in animal studies that re-epithelialization during wound healing was impaired in apolipoprotein (APO) E deficient mice suggesting a beneficial effect of pro-atherogenic lipoproteins on skin fibroblasts and granulation tissue formation, and a direct effect of APOE on wound healing [67]. Next to ALOX5AP as immune-modulator [68], several other factors of inflammation (e.g., ARHGD1/2, NAMPT, etc.) were detected in wound fluids. GDP-dissociation inhibitors of Rho proteins activates NAPDH oxidase (a direct cellular response to redox state) whereas a phosphoribosyltransferase (NAMPT), also known as a pre-B-cell colony enhancing factor 1, enables NAD^+^ biosynthesis. NAMPT functions as cytokine that promotes B cell maturation as well as inhibition of neutrophil apoptosis [36]. Strong immune modulators and clinical targets in hampered wound healing are chemokines and cytokines [69,70,71]. Despite their undeniable presence in acute wound healing as demonstrated a previous cohort [72], the limit of detection may be too low for detection via mass spectrometry or alternative approach such as targeted proteomics are potentially more suitable for their discovery.

A neutrophil proteomic signature was identified in wound fluids. Neutrophil elastase (ELANE) and cathepsin G (CTSG) are major components of neutrophil granules and participate in digestions of phagocytized microorganisms [73]. ELANE was one of the most abundant neutrophil proteins, and is essential in regulating microbial growth in wounds [74]. The presence of numerous proteins such as lactotransferin (LTF) or azurocidin 1 (AZU1) could be also shown in wound fluids of traumatic wounds in contrast to recently published proteomic studies [27] suggesting an effective impact of antimicrobial acting proteins on physiological wound healing. LTF had the highest abundance among all neutrophil-associated proteins. LTF aids in binding of nucleic acid and iron with a major role in destabilization of microbial membranes [75]. With all wounds ultimately healing properly indicating at least no pathological microbial burden after surgery, we can only speculate that the main role of LTF may not have been antimicrobial one. The second most abundant neutrophil-associated protein was SERPINB1, which acts as ELANE (also among most abundant proteins) inhibitor and is released by neutrophils themselves [76], underlining inflammatory fine-tuning in neutrophil products. Along similar lines, neutrophil gelatinase-associated lipocalin proteins (LCN) are involved in inflammation and detoxification processes caused by immune system activation in humans. Liver-derived PLG is known to be a critical regulator of cutaneous wound healing [77], and neutrophils are major cells targeting PLG [78]. We also identified BPI in highly abundant levels similar to a previous study where it was mildly associated with infection [79]. It is a potent protein antibiotic active against Gram-negative bacteria by binding to the lipopolysaccharide [80].

We identified a broad spectrum of extracellular matrix (ECM) proteins. Modulating the process of wound healing, ECM proteins bind numerous growth factors like TGF and release they after degradation of ECM proteins by proteolytic enzymes [81]. Not only the group members of collagen family is known to be strong associated with physiological wound healing but also the expression of fibrinogens and matrix metalloproteinases. They function as markers of formation of granulation tissue, re-epithelialization and ECM remodeling in dermal repair processes [81]. Proteins such as vitronectin, lumican, olfactomedin-4, a cartilage oligomeric matrix protein 2, and other glycoproteins (Table A2) are basic components of the newly formed provisional matrix at early wound healing stages and reflect—together with the presence of collagens—a physiological healing response. α2-macroglobulin (A2M), an anti-protease inhibiting proteinase, binds to and removes the active forms of gelatinases (e.g., MMP2 or 9) from the circulation via scavenger receptors on the phagocytes. A2M further inhibits plasminogen (PLG) and fibrolysis [82]. The vasodilator-stimulated phosphoprotein (VASP) and galectin 3 (LGALS3) play important roles in cell-cell adhesion and wound closure as well as in cell-matrix interactions, macrophage activation, and angiogenesis [83]. Moreover, calreticulin (CALR) and CD59 were identified, both present on human neutrophils [84]. In addition other 14-3-3 protein family members were detected in wound fluids, which mainly controls renewal of epithelium [85], and stimulation of epithelial cell migration after wounding [86]. Calcium binding proteins such as S100 factors are regulators of calcium homeostasis, inflammation, proliferation, cell cycle progress, and migration. They interact with a variety of target proteins on monocytes/macrophages, neutrophils, and lymphocytes [87]. S100A1 protein has been identified as novel regulator of endothelial angiogenesis suggestive to reflect pro-angiogenetic properties of this protein necessary for wound healing [88]. S100A4 presence is associated with processes of increased cell migration and transcriptional regulation of matrix metalloproteinases, e.g., MMP9, which was also found in our study. S100A6 has a role in cell response to different stressors such as heat shock proteins (e.g., HSP90). Among the S100 proteins, S100A8 and 9 are induced by pro-inflammatory stimuli in macrophages, dendritic cells, epithelial cells, and fibroblasts [89]. Both proteins were also found in wound exudates obtained from normal healing [27] corroborating our results. Additionally, it was recently shown that S100A8/9 overexpression in HaCaT keratinocytes increases NADPH oxidase activity and enhances ROS levels [87] underlining the concept of redox regulation during wound healing [64]. S100P reduces focal cell adhesion [90] emphasizing a support of cell migration. Moreover, the presence of S100A11 indicates a stimulation of keratinocyte cell growth by enhancing the level of several growth factors [91] and we have previously shown a redox regulation of several S100 proteins in human keratinocytes [92]. Based on our proteomic approach, the abundance of several annexins (ANX1, 2, 3, 4, 5, 11) was further demonstrated, which are linked to fibrinolysis, coagulation, inflammation and apoptosis [93], and to trafficking and organization of vesicles, exocytosis, endocytosis, calcium ion channel formation [94]. Trafficking and release of small vesicles, so-called microparticles, is important in neutrophil biology [95]. Neutrophil microparticles stain positive for CD15 [96]. Proteomic analysis of neutrophil-derived microparticles [97] was highly congruent with many targets found in wound fluids, among them annexin A1, A4, A5, and A11, AZU1, ELANE, MPO, BPI, B2M, CALR, CTSG, CTSZ, FCN-1, GRB2, LTF, LYZ, HSP70, HSP71, HSP90, MMP9, PRTN3, PRDX1, S100A9, and others, underlining the strong neutrophil signature in acute wound exudates. On annexins, Eming and colleagues validated its ability remove apoptotic cells to consolidate them as biomarkers to predict healing of traumatic, acute wounds [27].

Regarding chemical modifications, it is well known that oxidative as well as nitrosative modifications have a strong impact on several body functions, e.g., the immune response. Modifications observed by MS are known to be involved in various pathways in inflammation and wound healing. Methionine sulfoxide is the most common modification found by MS besides carbamidomethylation. It is described to attenuate the functions of NFκB and NFTA as well as indirectly influencing the T-cell receptor/CD3 signal transduction pathway [98], indicating its role in immune cell recruitment. In addition, oxidized cysteine residues were observed as the second most occurring modification. Cysteines, and their specific redox state, are key in recruiting leukocytes to freshly wounded tissue, e.g., by the Scr family kinase Lyn [99]. Both modifications can be introduced by hydroxyl radicals as well as hydroxyl radical-forming species, such as hydrogen peroxide, which is also known to be critical affecter of wound healing and immune cell recruitment [25,100,101]. Besides oxidative modification, the formation of nitrotyrosine was observed. Nitrotyrosine is widely regarded as a marker for inflammation [102], and nitrosated peptides can modulate the immune response when presented at MHC complexes [103]. Proteins incorporating nitrotyrosine are discussed to be formed by the impact of the reactive nitrogen species peroxynitrite [104]. Finally, a protein-*S*-nitrosocysteine was only observed in one instance. This specific PTM is supposed to be physiologically highly active in both innate and adaptive immune system regulation, e.g., by modulating toll-like receptor activity [105]. However, the S-NO moiety is comparably instable, and the occurrence in the wound fluids is likely being underestimated in mass spectrometric approaches. The various oxidative and nitrosative modifications observed are in good agreement with modifications typically associated with the immune response required to trigger wound healing. The redox-based signaling in immune system regulation both by RONS themselves as well as their resulting chemical modifications might be a prime target for clinical application [106]. A controlled regulation of the immune response might allow for faster wound healing with reduced chance of complications, e.g., the formation of chronic wounds. In this regard, first studies using topical applications of *S*-nitrosocysteine as a donor of the second messenger NO seem to be well-received and have the potential to enhance wound healing [107].

This study had limitations. Regarding the quantification of neutrophils from wound exudates, dead cells were excluded from the analysis only through forward scatter/side scatter profiles but not fluorescence dyes such as propidium iodide because immediate assaying after surgery was not always possible. Moreover, we did not compare surgical wound material with other wound types, such as chronic ulcers or burn wounds, limiting conclusions about potential therapeutic interventions with any of the targets identified. However, there was significant overlap of proteins identified with a previous study that had compared healing and non-healing wounds [27], validating our experimental approach. Nonetheless, this study is rather descriptive, allowing only speculating about the possible mechanistic role of proteins identified in human wound fluids.

In conclusion, we confidently identified hundreds of proteins present during healing of human wounds. Along with data from flow cytometry, this protein signature revealed a major role of neutrophils and their products in wound healing. Moreover, the proteomic study finding oxidoreductases and oxidative post-translational modifications provided evidence of redox control in wound healing. Both corroborates findings and hypothesis of previous studies but in addition resolves the molecular pattern to an extended degree. Together with future studies, this may help to resolve the central processes in human wound healing in physiology and pathology.

## 4. Materials and Methods

### 4.1. Wound Material

Wound exudates were collected onsite immediately after sponge removal in the surgical theater as described before [72]. Briefly, two sterile swabs were used to collect material of the wound area. Swabs were rinsed in tubes containing 1 mL of phosphate-buffered saline (PBS) on ice. Tubes were centrifuged, and the supernatant was collected and processed. Cell pellets were fixed with 1% paraformaldehyde until analysis. Wound sponge dressings obtained during surgery were immediately collected into tubes and stored at −80 °C until protein preparation. The sponges are made from polyurethane and are frequently used in vacuum assisted wound therapy. In total, proteins were isolated from 11 patients (Table 1). This study was approved by the local Greifswald ethics committee (BB 113/14, 12 January 2015).

### 4.2. Protein Preparation from Material in Wound Sponge Dressings

A small cube was cut from each dressing with roughly 5 mm edge length and each cube was weighted on a micro scale as swab loading differed strongly between patients. 50 µL RIPA buffer (1 mM EDTA, 0.5 mM EGTA, 1% (*v*/*v*) Triton X-100, 0.1% sodium deoxycholate, 0.1% (*w*/*v*) SDS, 140 mM NaCl, 1 mM PMSF, 10 mM Tris/HCl, pH 8) with protease and phosphatase inhibitors (Roche complete mini) was added to each swab per 10 mg wet weight and samples were incubated for 1 h on ice with periodic vortexing. Afterwards, samples were centrifuged at 4 °C for 1 h to remove cell debris and supernatants were decanted into fresh reagent tubes. Proteins were precipitated with acetone overnight and centrifuged for 1 h at 4 °C. To ease re-suspension of proteins, pellets were re-suspended in 500 mM Tris-HCl (pH 7.4), 10% (*w*/*v*) SDS, 5% (*w*/*v*) β-mercaptoethanol. Afterwards, protein concentrations were measured using a modified Bradford assay (RCDC, Bio-rad, Hercules, CA, USA). As each patient sample was differing strongly, “master” samples were generated by combining 10 µg protein of each sample together. In this way, a more comprehensive overview, which proteins would be present in high abundance in all patients, was possible.

Two master samples were generated independently and 30 µg protein each was loaded onto a precast Tris-Glycerine gel (Bio-rad) in duplicates. Gels were run at 125 V for about 90 min and stained/destained following standard procedures. Lanes containing samples were cut into 10 slices each. From this point on, all reagents and consumables were of LC/MS grade (e.g., LoBind, Eppendorf, Hamburg, Germany) Gel slices were destained using 400 µL washing solution (20 mM NH_4_HCO_3_ in 30% acetonitrile) on a shaker for 15 min. This procedure was repeated with fresh solution until slices were destained. Gel slices were dried in a vacuum centrifuge for about 35 min. Afterwards, proteins in the gel slices were reduced using 50 µL of 10 mM dithiothreitol at 60 °C for 1 h followed by an alkylation step with 50 µL of 50 mM iodoacetamide at 25 °C for 30 min. Afterwards, samples were washed twice with washing solution and supernatant was removed. Gel slices were again dried in a vacuum centrifuge for about 25 min (time adapted independently for each slice) prior to in-gel digestion using trypsin (10 µg/mL, sequencing grade, Promega, Wisconsin-Madison, WI, USA) in a volume adequate to cover the specific gel slices. Digestion was performed for 16 h at 22 °C.

After digestion, peptides were extracted from the gel slices by consecutively using 50 µL of 5% formic acid, 50 µL acetonitrile, and 100 µL acetonitrile. After each solvent addition, samples were toughly vortexted for 15 min, centrifuged for 1 min and supernatants pooled in a fresh reagent tube. Afterwards, the resulting 200 µL were reduced to about 10 µL by vacuum centrifugation and 20 µL of *A. dest.* added. Samples were stored at −80 °C until measurement.

### 4.3. Mass Spectrometry

Samples were measured in a randomized order. The LC-MS system consisted of an Ultimate 3000 (Dionex, Sunnyvale, CA, USA) nanoLC using an Acclaim PepMap 100 guard column with an Acclaim PepMap RSLC column (15 cm, 75 µM ID, both heated to 40 °C) and a QExactive (Thermo Scientific, Waltham, MA, USA) MS with a Nanospray Flex (ThermoFischer Scientific, Dreieich, Germany) source using steel emitters. The gradient used for the LC was as followed with the solvents A (*A. dest*, 0.1% acetic acid) and B (acetonitrile, 0.1% acetic acid): 0–4 min: 2% B, 4–65 min linear ramp to 35% B, 65–75 min linear ramp to 50% B, 75–80 min 50% B, 80–81 min linear ramp to 80% B, 81–85 min 80% B, 85–86 min linear ramp to 2% B, 86–100 min 2% B. Total flow was 300 nL/min. Calibration was performed on a daily basis using Pierce™ LTQ Velos ESI Positive Ion Calibration Solution (Thermo Scientific). QExactive measurements were conducted in positive mode in Full MS → dd-MS^2^ mode. For survey scans, 70,000 resolution in the range of 300 to 1650 *m*/*z* was used with an AGC target of 10^6^ and an ion time of 120 ms. For the Top10 MS/MS scans, a 17,500 resolution with an AGC target of 2 × 10^5^ and a stepped NCE of 27.5. Exclusion time for Top10 picking was 30 s, meaning that the 10 highest signals in a survey scan at any given time were fragmented and MS/MS spectra collected. Afterwards, these exact signals were ignored for determination of the highest signals for the next 30 s.

### 4.4. Data Analysis

Raw data were analyzed using Proteome Discoverer 2.0 (Thermo Scientific). All 10 gel slices from one lane, which were measured independently, were defined as fractions of a single experiment, resulting in four meta-samples. They were searched against the reviewed human proteome (Uniprot UP000005640) with a FDR of 0.01 (strict) and 0.05 (medium) for both peptides and PSMs. At least 2 unique peptides had to be present for strict annotation. For relative quantification, number of peptide spectral matches normalized by protein length, was used as given by the softwate (#PSM). The following flexible modifications were included: carbamidomethylation at C, single oxidation at C & M, trioxidation at C & W, and nitration at Y. Carbamidomethylation was not set as a fixed modification as wound exposure to ambient conditions might result in oxidative modifications at cysteines. These modifications might not be properly reduced by DTT in the workflow. Results were tested against a mock-up database consisting of randomly generated peptides to determine the false discovery rate (FDR). Annotation procedure was attuned to keep FDR below the 0.05 (medium) and 0.01 (strict) thresholds. Additional information were retrieved using free online PANTHER analysis tools (Figure 1).

### 4.5. Analysis of Manually Collected Wound Material

To process cell pellets, the fixative was washed off, and cell were stained either with anti-myeloperoxidase (MPO) PE antibodies and 4′,6-diamidin-2-phenylindol (DAPI) to delineate DNA-MPO aggregates, or with antibodies targeted against anti-CD45 PE-Cy7, CD66b PerCP-Cy5.5, and CD16 PE-Dazzle (all BioLegend, San Diego, CA, USA). Cells were washed, and analyzed by multicolor flow cytometry (Beckman-Coulter, Brea, CA, USA). Supernatants obtained after centrifugation of swap material dissolved in PBS were investigated in several ways. To analyze microparticle and their origin, supernatant was centrifuged at 14,000× *g*, washed, stained with anti-CD15 APC (BioLegend) and bio-maleimide (BODIPY FL *N*-(2-Aminoethyl; Life Technologies, Carlsbad, CA, USA), washed, and analyzed by flow cytometry (Beckman-Coulter). Beads were utilized to set up the cytometer and to retrieve maximum sensitivity triggering over the side scatter as previously described [97]. Flow analysis was performed with Kaluza software 1.5a (Beckman-Coulter). Graphs were made using prism 7.03 (Graphpad software). Sytox green (Thermo Scientific) was added supernatants, and the DNA concentration was quantified against a DNA standard curve (Thermo Scientific). Fluorescence was read using a microplate reader (Tecan, Männedorf, Switzerland) at λ_ex_ 485 nm and λ_em_ 535 nm. In a similar fashion, fluorescence of H_2_-DCF (Sigma, Taufkirchen, Germany) added the samples was determined to investigate presence of oxidants and oxidase activity.

## Figures and Tables

**Table 1:** Patients enrolled in this study.

Cohort Feature	Value
Number of Patients	11
Median patient age (years ± S.E.)	54 ± 4
Sex (m/f)	9/2
Wound type	11 trauma wounds (1 thoracic, 3 upper extremity, 7 lower extremity)
Wound healing response	11/11
Median time to wound healing (days ± S.E.)	21 ± 4

**Table 2:** Oxidoreductases in wound fluids.

Protein ID	Acronym	Protein Name	Protein Class
P04040	CAT	Catalase	Peroxidase
Q06830	Prdx1	Peroxiredoxin 1	Peroxidase
P32119	Prdx2	Peroxiredoxin 2	Peroxidase
P30041	Prdx6	Peroxiredoxin 6	Peroxidase
P05164-2	MPO	Isoform H14 of Myeloperoxidase	Peroxidase
P00441	SOD1	Superoxide dismutase 1	Oxidoreductase
P00403	MT-CO2	Cytochrome C oxidase 2	Oxidoreductase
P16083	NQO2	Ribosyldihydronicotinamide dehydrogenase	Oxidoreductase
O95571	ETHE1	Persulfide dioxygenase	Oxidoreductase
P07195	LDHB	l-lactate dehydrogenase β	Dehydrogenase
P40926	MDH2	Malate dehydrogenase 2	Dehydrogenase
P30043	BLVRB	Flavin reductase (NADPH)	Reductase
P20292	ALOX5AP	Arachidonate 5-lipoxygenase activating protein	Transferase

**Table 3:** Signaling and immune modulators, chaperones, and heat shock proteins in wound fluids. GDP: Guanosine-5′-triphosphate.

Protein ID	Acronym	Protein Name
*Calmodulin-signaling molecules*		
P26447	S100A4	Protein S100 A4
P06703	S100A6	Protein S100 A6
P05109	S100A8	Protein S100 A8
P06702	S100A9	Protein S100 A9
P31949	S100A11	Protein S100 A11
P25815	S100P	Protein S100 P
*Chaperone and heat shock proteins*		
P31946	YWHAB	14-3-3 protein β/α
P61981	YWHAG	14-3-3 protein γ
P27348	YWHAH	14-3-3 protein τ
Q04917	YWHAQ	14-3-3 protein ε
P63104	YWHAZ	14-3-3 protein ζ
P27797	CALR	calreticulin
P14625	HSP90B1	Endoplasmin
P0DMV9	HSPA1B	Heat shock 70 kDa protein 1B
P11142	HSPA8	Heat shock cognate 71 kDa protein
P11142-2	HSPA8	Isoform 2 of Heat shock cognate 71 kDa protein
P0DMV8-2	HSPA1A	Isoform 2 of Heat shock 70 kDa protein 1A
*Immune modulators*		
P01023	A2M	α2-macroglobulin
P43490	NAMPT	Nicotinamide phosphoribosyltransferase
P52565	ARHGD	Rho GDP-dissociation inhibitor 1
P52566	ARHGD	Rho GDP-dissociation inhibitor 2
P04083	ANXA1	Annexin A1
P07355	ANXA2	Annexin A2
P12429	ANXA3	Annexin A3
P09525	ANXA4	Annexin A4
P09525-2	ANXA4	Annexin A4 Isoform 2
P08758	ANXA5	Annexin A5
P50995	ANXA11	Annexin A11
P50995-2	ANXA11	Annexin A11 Isoform 2
P20292	ALOX5AP	Arachidonate 5-lipoxygenase activating protein

**Table 4:** Neutrophil- and leukocyte-associated factors in wound fluids.

Protein ID	Acronym	Protein Name
P20160	AZU1	Azurocidin
P02788	LTF	Lactotransferrin
P02788-2	LTF	Isoform δLf of Lactotransferrin
P17213	BPI	Bactericidal permeability-increasing protein
P08311	CTSG	Cathepsin G
Q9UBR2	CTSZ	Cathepsin Z
P00747	PLG	Plasminogen
P61626	LYZ	Lysozyme
P08246	ELANE	Neutrophil elastase
P30740	SERPINB1	Leukocyte elastase inhibitor
P30740-2	SERPINB1-2	Leukocyte elastase inhibitor isoform 2
P80188	LCN2	Neutrophil gelatinase-associated lipocalin
P80188-2	LCN2	Isoform 2 of Neutrophil gelatinase-associated lipocalin
Q15582	TGFB1	Transforming growth factor B1

**Table 5:** Extracellular matrix proteins in wound fluids.

Protein ID	Acronym	Protein Name
P02671	FGA	Fibrinogen α chain
P02675	FGB	Fibrinogen β chain
P02679	FGG	Fibrinogen γ chain
P02671-2	FGA2	Isoform 2 of Fibrinogen α chain
P02679-2	FGGA	Isoform γ A of Fibrinogen γ chain
P02452	COL1A1	Collagen α-1(I) chain
P08123	COL1A2	Collagen α-2(I) chain
P20908	COL5A1	Collagen α-1(V) chain
P12109	COL6A1	Collagen α-1(VI) chain
O00602	FCN-1	Ficolin 1
P51884	LUM	Lumican
P04004	VTN	Vitronectin
Q6UX06	OLFM4	Olfactomedin-4
P49747-2	COMP2	Cartilage oligomeric matrix protein 2
P17931	LGALS3	Galectin 3
P50552	VASP	Vasodilator-stimulated phosphoprotein

**Table 6:** Proteinases and peptidases in wound fluids.

Protein ID	Acronym	Protein Name
P13798	ACPH	Acylamino-acid-releasing enzyme
P15144	AMPN	Aminopeptidase N
P15169	CBPN	Carboxypeptidase N catalytic chain
P42574	CASP3	Caspase-3
P08311	CATG	Cathepsin G
Q9UBR2	CATZ	Cathepsin Z
Q9NZP8	C1RL	Complement C1r subcomponent-like protein
P09871	C1S	Complement C1s subcomponent
P00751	CFAB	Complement factor B
P05156	CFAI	Complement factor I
P02788	TRFL	Lactotransferrin
P14780	MMP9	Matrix metalloproteinase-9
P24158	PRTN3	Myeloblastin
P08246	ELNE	Neutrophil elastase
P00747	PLMN	Plasminogen
P25789	PSA4	Proteasome subunit α type-4
P28066	PSA5	Proteasome subunit α type-5
P20618	PSB1	Proteasome subunit β type-1
P40306	PSB10	Proteasome subunit β type-10
P28070	PSB4	Proteasome subunit β type-4
P28072	PSB6	Proteasome subunit β type-6
Q99436	PSB7	Proteasome subunit β type-7
P00734	THRB	Prothrombin

## Appendix A

**Table A1:** Apolipoproteins, glycoproteins, and protease inhibitors in wound fluids.

Acronym	Protein Name	Acronym
P08519	LPA	Apolipoprotein(a)
P02647	APOA1	Apolipoprotein A-I
P02652	APOA2	Apolipoprotein A-II
P02656	APOC3	Apolipoprotein C-III
P06727	APOA4	Apolipoprotein A-IV
P04114	APOB	Apolipoprotein B-100
P05090	APOD	Apolipoprotein D
P02649	APOE	Apolipoprotein E
O14791-3	APOL1	Isoform 3 of Apolipoprotein L1
P04217	A1BG	Alpha-1B-glycoprotein
P04217-2	A1BG-2	Isoform 2 of Alpha-1B-glycoprotein
P02749	APOH	Beta-2-glycoprotein 1
P13987	CD59	CD59 glycoprotein
P04196	HRG	Histidine-rich glycoprotein
P11279	LAMP1	Lysosome-associated membrane glycoprotein
P02750	LRG1	Leucine-rich α-2-glycoprotein
P02763	ORM1	Alpha-1-acid glycoprotein
P02765	ORM2	Alpha-2-acid glycoprotein
P25311	AZGP1	Zinc-α-2-glycoprotein
P01009	SERPINA1	Alpha-1-antitrypsin
P01009-2	SERPINA1-2	Isoform 2 of α-1-antitrypsin
P01009-3	SERPINA1-3	Isoform 3 of α-1-antitrypsin
P01011	SERPINA3	Alpha-1-antichymotrypsin
P01011-2	SERPINA3-2	Isoform 2 of α-1-antichymotrypsin
P05154	SERPINA5	Plasma serine protease inhibitor
P08185	SERPINA6	Corticosteroid-binding globulin
P35237	SERPB6	Serpin B6
P05546	SERPIND1	Heparin cofactor 2
P08670	SERPINF1	Pigment epithelium-derived factor
P08697	SERPINF2	Alpha-2-antiplasmin
P08697-2	SERPINF2-2	Isoform 2 of α-2-antiplasmin
P00734	F2	Prothrombin
P01008	SERPINC3	Antithrombin-III

**Table A2:** Complement components in wound fluids.

Acronym	Protein Name	Acronym
P01024	C3	Complement C3
P0C0L5	C4B	Complement C4-B
P01031	C5	Complement C5
P13671	C6	Complement component C6
P07360	C8G	Complement component C8 γ chain
P02748	C9	Complement component C9
P00751	CFB	Complement factor B
P00751-2	CFB	Isoform 2 of Complement factor B
P08603	CFH	Complement factor H
P36980	CFHR2	Complement factor H-related protein 2
P05156	CFI	Complement factor I
Q9NZP8	C1RL	Complement C1r subcomponent-like protein
P02746	C1QB	Complement C1q subcomponent subunit B
P02747	C1QC	Complement C1q subcomponent subunit C
P09871	C1S	Complement C1s subcomponent

**Table A3:** PTMs found in wound fluids (high confidence hits only).

Protein ID	Acronym	Protein Name	PTMs ^a^
P01834	IGKC	Immunoglobulin kappa constant	Trioxidation [C87]
P15814	IGLL1	Immunoglobulin lambda-like polypeptide 1	Trioxidation [C194]; Oxidation [M197]
Q04695	KRT17	Keratin, type I cytoskeletal 17	Oxidation [M88]
P30043	BLVRB	Flavin reductase (NADPH)	Oxidation [M87];
P02549	SPTA1	Spectrin α chain, erythrocytic 1	Oxidation [M647; M881]
P02549-2	SPTA1	Isoform 2 of Spectrin α chain, erythrocytic	Oxidation [M647; M881]
P11142	HSPA8	Heat shock cognate 71 kDa protein	Oxidation [M61]
P0DMV9	HSPA1B	Heat shock 70 kDa protein 1B	Oxidation [M549]
P0DMV8-2	HSPA1A	Isoform 2 of Heat shock 70 kDa protein 1A	Oxidation [M494]
Q14624	ITIH4	Inter-α-trypsin inhibitor heavy chain H4	Oxidation [M491]
Q562R1	ACTBL2	Beta-actin-like protein 2	Oxidation [M45; M48; M191];
P26038	MSN	Moesin	Oxidation [M433; M451]
P12429	ANXA3	Annexin A3	Oxidation [M40]
P01871	IGHM	Immunoglobulin heavy constant mu	Oxidation [M383];
P01871-2	IGHM	Isoform 2 of Immunoglobulin heavy constant mu	Oxidation [M383];
P04004	VTN	Vitronectin	Oxidation [M350];
P35542	SAA4	Serum amyloid A-4 protein	Oxidation [M35]
P30101	PDIA3	Protein disulfide-isomerase A3	Oxidation [M338]
P06727	APOA4	Apolipoprotein A-IV	Oxidation [M322]
P30740	SERPINB1	Leukocyte elastase inhibitor	Oxidation [M307]
P04114	APOB	Apolipoprotein B-100	Oxidation [M306; M3007; M3421]
P04264	KRT1	Keratin, type II cytoskeletal 1	Oxidation [M296];
P35237	SERPINB6	Serpin B6	Oxidation [M291]
P40121	CAPG	Macrophage-capping protein	Oxidation [M261]
P02671	FGA	Fibrinogen α chain	Oxidation [M259]
P02671-2	FGA	Isoform 2 of Fibrinogen α chain	Oxidation [M259]
P40121-2	CAPG	Isoform 2 of Macrophage-capping protein	Oxidation [M246]
P61981	YWHAG	14-3-3 protein γ	Oxidation [M23; M27]
P02790	HPX	Hemopexin	Oxidation [M229; M375; M409];
P47756-2	CAPZB	Isoform 2 of F-actin-capping protein subunit β	Oxidation [M220]
Q06830	PRDX1	Peroxiredoxin-1	Oxidation [M21]
P27105	STOM	Erythrocyte band 7 integral membrane protein	Oxidation [M207; M228]
O15145	ARPC3	Actin-related protein 2/3 complex subunit 3	Oxidation [M19]
P01860	IGHG3	Immunoglobulin heavy constant γ 3	Oxidation [M182; M288]; Trioxidation [C27; C297];
P09525	ANXA4	Annexin A4	Oxidation [M17]
P19823	ITIH2	Inter-α-trypsin inhibitor heavy chain H2	Oxidation [M162]
P35527	KRT9	Keratin, type I cytoskeletal 9	Oxidation [M157; M234; M245; M269]
P08670	VIM	Vimentin	Oxidation [M154]
P13645	KRT10	Keratin, type I cytoskeletal 10	Oxidation [M150]
P00915	CA1	Carbonic anhydrase 1	Oxidation [M149; M242]
P01857	IGHG1	Immunoglobulin heavy constant γ 1	Oxidation [M135]; Trioxidation [C27; C250]; Nitro [Y161]
P01861	IGHG4	Immunoglobulin heavy constant γ 4	Oxidation [M132]; Trioxidation [C247];
P01859	IGHG2	Immunoglobulin heavy constant γ 2	Oxidation [M131; M237]; Trioxidation [C246];
P02787	TF	Serotransferrin	Oxidation [M128; M332; C374]; Trioxidation [C374]
P08779	KRT16	Keratin, type I cytoskeletal 16	Oxidation [M121];
P02533	KRT14	Keratin, type I cytoskeletal 14	Oxidation [M119];
P02647	APOA1	Apolipoprotein A-I	Oxidation [M110; M136; M172]
P37837	TALDO1	Transaldolase	Oxidation [M11]
P02679-2	FGG	Isoform γ-A of Fibrinogen γ chain	Oxidation [M104; M290];
P02679	FGG	Fibrinogen γ chain	Oxidation [M104; M290];
P02792	FTL	Ferritin light chain	Oxidation [M101]
A0M8Q6	IGLC7	Immunoglobulin lambda constant 7	Oxidation [C87]; Nitrosyl [C87]; Nitro [Y85]
A0A0C4DH72	IGKV1	Immunoglobulin kappa variable 1–6	Oxidation [C22; M26]
B9A064	IGLL5	Immunoglobulin lambda-like polypeptide 5	Oxidation [C195]; Trioxidation [C195]
P00450	CP	Ceruloplasmin	Nitro [Y471]; Oxidation [M538]
P68871	HBB	Hemoglobin subunit β	Nitro [Y36]; Oxidation [M56];
P02042	HBD	Hemoglobin subunit δ	Nitro [Y36]
P00738	HP	Haptoglobin	Nitro [Y280]; Oxidation [M263; M300; M343]
P69905	HBA1	Hemoglobin subunit α	Nitro [Y25]; Oxidation [M33]
P00738-2	HP	Isoform 2 of Haptoglobin	Nitro [Y221]; Oxidation [M204; M241; M284]
P01009	SERPINA1	Alpha-1-antitrypsin	Nitro [Y184]; Oxidation [M409]
P01009-3	SERPINA1	Isoform 3 of Alpha-1-antitrypsin	Nitro [Y184]
P01009-2	SERPINA1	Isoform 2 of Alpha-1-antitrypsin	Nitro [Y184]
P49913	CAMP	Cathelicidin antimicrobial peptide	Nitro [Y56]; Oxidation [M109]
P02768	ALB	Serum albumin	Oxidation [M111; C125; C289; M353; C500; C501; M572]; Trioxidation [C125; C192; C224; C289; C302; C303; C500; C501; C591]; Nitro [Y164; Y287]; Nitrosyl [C289]
P02768-3	ALB	Isoform 3 of Serum albumin	Oxidation [M111; C125; C287; C288; M359]; Trioxidation [C125; C287; C288; C378]
P02768-2	ALB	Isoform 2 of Serum albumin	Oxidation [C97; M161; C308; C309; M380]; Trioxidation [C97; C110; C111; C308; C309; C399]; Nitro [Y95]; Nitrosyl [C97]
P0C0L5	C4B	Complement C4-B	Oxidation [M393]
P01023	A2M	Alpha-2-macroglobulin	Oxidation [M713; M1378]
P01024	C3	Complement C3	Oxidation [M164; M495; M581; M990; M1347; M1384]
P08603	CFH	Complement factor H	Oxidation [M1174]
P02788	LTF	Lactotransferrin	Oxidation [C28; M411]; Trioxidation [C28]
P02675	FGB	Fibrinogen β chain	Oxidation [M254; M272]
P02788-2	LTF	Isoform δLf of Lactotransferrin	Oxidation [M367]
P02763	ORM1	Alpha-1-acid glycoprotein 1	Nitro [Y175]
P04406	GAPDH	Glyceraldehyde-3-phosphate dehydrogenase	Oxidation [M328; M331]
P07738	BPGM	Bisphosphoglycerate mutase	Oxidation [M103]
P13796	LCP1	Plastin-2	Oxidation [M166]
P04406-2	GAPDH	Isoform 2 of Glyceraldehyde-3-phosphate dehydrogenase	Oxidation [M286; M289]
P07738	BPGM	Bisphosphoglycerate mutase	Oxidation [M103]
P13796	LCP1	Plastin-2	Oxidation [M166]

^a^ Carbamidomethylations were excluded due to their introduction during sample preparation and their ubiquitous presence.
